# Highly-Optimized Radar-Based Gesture Recognition System with Depthwise Expansion Module

**DOI:** 10.3390/s21217298

**Published:** 2021-11-02

**Authors:** Mateusz Chmurski, Gianfranco Mauro, Avik Santra, Mariusz Zubert, Gökberk Dagasan

**Affiliations:** 1 Infineon Technologies AG, 85579 Neubiberg, Germany; Gianfranco.mauro@infineon.com (G.M.); avik.santra@infineon.com (A.S.); mariusz.zubert@p.lodz.pl (M.Z.); goekberk.dagasan@infineon.com (G.D.); 2 Department of Microelectronics and Computer Science, Lodz University of Technology, 90924 Lodz, Poland; 3 Department of Electronic and Computer Technology, University of Granada, Avenida de Fuente Nueva s/n, 18071 Granada, Spain

**Keywords:** edge computing, Edge TPU, optimization, quantization, FMCW, radar, deep learning, neural networks

## Abstract

The increasing integration of technology in our daily lives demands the development of more convenient human–computer interaction (HCI) methods. Most of the current hand-based HCI strategies exhibit various limitations, e.g., sensibility to variable lighting conditions and limitations on the operating environment. Further, the deployment of such systems is often not performed in resource-constrained contexts. Inspired by the MobileNetV1 deep learning network, this paper presents a novel hand gesture recognition system based on frequency-modulated continuous wave (FMCW) radar, exhibiting a higher recognition accuracy in comparison to the state-of-the-art systems. First of all, the paper introduces a method to simplify radar preprocessing while preserving the main information of the performed gestures. Then, a deep neural classifier with the novel Depthwise Expansion Module based on the depthwise separable convolutions is presented. The introduced classifier is optimized and deployed on the Coral Edge TPU board. The system defines and adopts eight different hand gestures performed by five users, offering a classification accuracy of 98.13% while operating in a low-power and resource-constrained environment.

## 1. Introduction

In recent years, computing technology has become an intrinsic part of our daily lives, and automation is becoming inevitable [1]. As a result, the existing HCI methods, such as keyboard and mouse, are being replaced by more intuitive solutions, e.g., hand gesture recognition systems [2,3]. Conventional HCI approaches mainly employ optical sensors (e.g., RGB and ToF cameras), speech recognizing sensors, and wearable devices [4,5,6,7,8,9,10,11,12,13,14,15]. Optical sensors are being commonly used for motion sensing and gesture recognition. Optical-based gesture recognition frameworks are highly accurate but are, in general, environment dependent [15,16]. In such systems, lightning conditions negatively affect the overall system performance. Privacy concern is another downside of camera-based gesture recognition. Speech-based HCI may provide an interactive environment. However, the tonal and physical variations, e.g., background noise, drastically influence the overall system accuracy [11,12,13,17]. To deal with these problems, wearable devices have been proposed to improve the overall system’s performance [18,19]. The need to wear a device all the time may not be an ideal solution for many users. Unlike optical sensors and wearable devices, radar-based gesture recognition techniques may overcome those limitations [20]. Radar sensors are not affected by variable lighting conditions and further, when adequately employed, do not lead to privacy concerns. In addition, radars can provide a touchless environment for capturing gestures, as a result, users do not have to wear additional hardware [20,21].

Another concern of the contemporary HCI frameworks is their power [22,23,24,25,26]. The typical operation of HCI frameworks is based on the analysis of the spatial–temporal relations between consecutive frames utilizing the deep learning methods, e.g., 3D convolutional neural networks (CNN3D), long short-term memory (LSTM), and Recurrent Neural Networks (RNN) [27,28,29,30]. This analysis is a computationally complex task, which prevents deployment on resource-constrained devices [23,24,25,26,31,32,33,34,35,36,37,38,39].

In recent years, researches carried out by numerous teams in R&D centers set out the path, which led to the development of such topologies as AlexNet [40], VGGNet [41], and ResNet [42]. These topologies achieved tremendous success in the field of computer vision. They can learn the deep representation of the data and solve sophisticated tasks [43,44]. However, the high accuracy achieved by the deep learning models comes at the expense of increased computational and memory requirements for both the training and inference phases. Training the deep learning model is memory and computationally expensive due to the potentially high dimensionality of the input data (e.g., a high-resolution image) and the millions of computations that need to be performed. High resource consumption is the main bottleneck of the deep learning methods, especially when the application aims to deploy computationally complex algorithms on the less powerful edge computing device [45].

The latest developments in deep learning are leading the research focus towards the development of optimization methods and the deployment on edge devices. According to Ericsson [46], 45% of global internet congestion in 2021 is going to be produced by IoT (Internet of Things) devices, which confirms the need for in-depth research in this direction. The fundamental principle of edge computing is shifting the computation and communication resources from the cloud to the edge of networks [47], to avoid communication latency, provide a privacy protection capability, and enable a faster response to the end users. Therefore, the necessity to optimize the deep learning models for the deployment on the edge of the network is a relevant aspect to improve overall system performance [48].

Neural network optimization methods, including both architectural design and post-training adaptations, enable developers to transform complex models into streamlined implementations [49,50]. Architectural optimization methods are often conceived for the deployment on resource-constrained hardware. They are realized by replacing the traditional convolutions with depthwise separable convolutions, squeezing the output of the convolutional layer using 1 × 1 convolution, or splitting the kernels into horizontal and vertical components, as in the case of spatially separable convolution [51,52]. These strategies have been used in several renowned topologies such as MobileNetV1 [51], SqueezeNet [53], MixNet [54], and GoogleLeNet [55]. Other optimization methods involve, e.g., hyperparameter configuration [56] and automatic architecture search [57]. Post-training model adaptations involve pruning [58,59,60], quantization [61,62,63], and format optimization.

Another important aspect lies in the input data structure, e.g., high-dimensional images, which directly affect the number of computations and the required memory allocation.

Recent radar-based gesture recognition classifiers are linear structures built by stacking the convolutional layers or using recurrent structures, thereby increasing the algorithm’s model size, latency, and computational complexity. However, researchers have never paid attention to applying more advanced topologies with lightweight building blocks on radar data. Andrew et al. [51] present the class of efficient models called MobileNets for mobile and embedded vision applications. This work has introduced the concept of depthwise-separable convolutions, which is a form of factorized operation that separates a standard convolution kernel into depthwise and 1 × 1 pointwise convolution kernels. In this algorithm, the depthwise convolution applies a single convolutional filter to each input channel; then, pointwise convolution uses a 1 × 1 convolution to combine the outputs of the depthwise convolution.

Specific techniques for the reduction of network size are shrinking, factorizing, or compressing the pretrained networks [64,65]. Another commonly employed method is distillation [66], which makes use of one or more large networks to teach a smaller network how to achieve comparable results. Another approach, introduced in the second generation of MobileNet, relies on applying the residual connections between consecutive layers. Rather than simply stacking the layers linearly, MobileNetV2 employs a novel building block known as inverted residuals with a linear bottleneck [52]. This module takes as input a low-dimensional compressed representation of data which is first expanded to high dimension and filtered with a lightweight depthwise convolution. The extracted features are then projected back to a low-dimensional representation with a linear convolution. The proposed module is suitable for edge devices, decreasing the number of parameters and memory footprint needed during the inference time. MobileNets have found several applications, particularly in image classification, object detection, and semantic segmentation. This paper aims to design the dedicated topology for hand gesture recognition based on the MobileNetV1 architecture employing the ideas coming from MobileNetV2, i.e., increasing and decreasing the number of feature maps.

Inspired by the extensive usage of MobileNet architectures in problems related to image classification, this work presents a dedicated topology within a novel deep learning module—***Depthwise Expansion Module***. The proposed solution utilizes the depthwise convolutions, followed by the standard CNN2D performing a feature embedding. The depth of the topology is regulated by α parameter, where α∈{0.25,0.50,0.75,1.00}. The system classifies the FMCW radar signal representing eight gestures. The main objective is to obtain higher recognition accuracy than state-of-the-art frameworks for radars, by simultaneously reducing the number of parameters, model size, and inference time. The main modifications compared to the original MobileNetV1 implementation include the replacement of convolutional layers by linearly increasing the number of feature maps through the ***Depthwise Expansion Module*** and the usage of fully connected layers in the place of the global average pooling layer. Moreover, we have adapted the size of the input tensor to our data and obtained higher recognition accuracy than the state-of-the-art frameworks. In the proposed framework, the signal from the FMCW radar has been transformed into the compressed representation to avoid the usage of ineffective neural network operators. The gesture vocabulary comprises eight gestures. The data collection setup consists of Raspberry Pi4, tripod, and an Infineon BGT60TR13C radar sensor, while the inference setup is built of Coral Edge TPU, tripod, and an Infineon BGT60TR13C radar board. The acquired samples of each gesture have been preprocessed and then transformed into a 3D tensor, including the range time, velocity time and azimuth time maps. After data preprocessing, the model has been trained, optimized, and deployed on the Coral Edge TPU board.

The main contributions of this paper are as follows:

1. We present a novel building block—Depthwise Expansion Module. To the best of our knowledge, this type of building block has never been proposed in the field of radar-based gesture recognition.
2. We deploy and test our algorithm on Edge TPU, proposing the 8-bit algorithm implementation. As far as we are aware, we propose the first radar-based gesture recognition classifier, which is optimized and deployed on the Coral Edge TPU board.
3. We propose a signal processing pipeline that allows a compressed data representation of the FMCW radar signal.

## 2. Related Works

In the first part of this chapter, we analyze the gesture recognition methods based on radar. In the next part, we focus on gesture techniques based on alternative modalities, i.e., RGB, depth, and infrared sensors.

In this work, we use the frequency-modulated continuous wave (FMCW) radar sensor manufactured by Infineon AG. The FMCW modulation technique has found many applications, e.g., people counting [67,68], vital sign detection [69,70], and gesture recognition [20]. Recently, the FMCW radars are also finding applications in the automotive industry [71,72,73]. High-end cars already employ radars in the context of parking assistance and lane departure warnings. Currently, there is growing interest in applying touchless sensors in many devices.

An algorithm called Long Recurrent All Convolutional Neural Network (LRACNN) employing FMCW radar data has been proposed by Hazra et al. [27] with the aim of hand gesture recognition. The algorithm utilizes a time-distributed layer wrapper and the same set of convolutional layers at each input time step. The feature vector, extracted by the time-distributed layer, is fed to an LSTM layer for the temporal feature modeling. The extracted features are then input into a fully connected layer for final classification and marked prediction accuracy of 94.34% is achieved. The proposed algorithm employs a high amount of resources, and therefore could not be supported by most edge computing devices. Consequently, the algorithm would not be deployable on highly-constrained devices such as Intel Neural Compute Stick 2 (NCS 2) or Coral Edge TPU.

Zhang et al. [74] presented a hand gesture recognition methodology based on the CNN3D and LSTM layers. The CNN3D is used for spatial–temporal feature extraction while the LSTM is employed for the global temporal feature modeling. This method exhibits a satisfactory recognition capability of 96.0%. However, the memory footprint and the number of computations increase by the combination of CNN3D with LSTM, leading to a solution hardly deployable on resource-constrained hardware.

Ahmed et al. [75] propose a hand gesture recognition system that uses an impulse radio ultra-wideband (IR-UWB) radar and a classifier based on nine inception modules. The results of this work exhibit higher classification accuracy than most of the state-of-the-art solutions. However, the complex signal processing scheme and intricate structure of the classifier imply high resource consumption.

Hazra et al. [28] introduced a hand gesture recognition classifier based on CNN3D feature embedding. This work matches CNN3D with triplet loss to learn the embedded feature vectors. The extracted features are the input of a k-NN (k-Nearest Neighbour) algorithm for the final inference. This approach achieves good classification accuracy, while it exhibits similar constraints to the ones mentioned above.

Molchanov et al. [76] introduced a radar-based gesture sensing system that employs a specific signal processing methodology for the generation of the range-Doppler maps (RDMs) and angle maps. The angle information is used to synchronize the radar with the ToF camera in the perspective of a multisensor system for automotive applications. A dedicated CNN3D classifier achieves satisfactory classification accuracy. However, the proposed signal processing scheme and CNN3D classifier are huge limitations for resource-constrained edge deployment.

Lien et al. [77] have taken the initial steps to investigate the radar as a new gesture sensing modality. This work introduces the whole gesture processing pipeline (i.e., data collection, digital signal preprocessing, signal transformations, feature extraction, and training the classifier). The pipeline conveys a low-dimensional features solution for the implementation of a possibly simplified prediction through Random Forest Classifier (RFC). The proposed approach has been tested on two energy-efficient platforms, i.e., Raspberry Pi2 and Qualcomm Snapdragon 400.

Chmurski et al. [78] paved the path for deploying a radar-based gesture recognition system on a resource-constrained device such as Raspberry Pi. In this work, an optimized signal processing pipeline using continuous wavelet transform (CWT) maps is presented. The model topology is based on a time-distributed layer wrapper that applies the same set of convolutional layers to each timestamp, achieving a good classification accuracy of 95.05%. However, the proposed signal processing and classifier cause high resource consumption as end-to-end system latency is around 1 s, not enabling real-time system operation. In [79], the previously proposed family of gesture recognition classifiers is optimized and deployed on the Intel Neural Compute Stick 2 (Intel NCS 2). This work forms the foundation of further research in this direction.

The alternative approaches for gesture recognition include the usage of different sensor modalities. In [23], D’Eusanio et al. propose the transformer-based neural network with a self-attention mechanism, weighting the importance of each part of the input data. The proposed classifier is built from the ResNet-18-base visual feature extractor. In the next step, the extracted features are processed by the temporal feature analyzer, and finally, the classification is performed. The proposed classifier has been tested on two widely-known gesture recognition datasets, i.e., Nvidia Dynamic Hand Gesture dataset [24] and Briareo dataset [80], with different data modalities, i.e., RGB, depth, infrared, and normals. In the best case, the proposed algorithm achieves good classification results, i.e., 87.6% and 97.2% for the Nvidia and Briareo datasets, respectively. However, it exhibits some limitations concerning the deployment on resource-constrained devices, i.e., in the case of four data modalities (RGB, depth, infrared, and normals) the classifier has 97.2 M parameters, and it requires 5.3 GB of VRAM memory.

Another approach has been proposed by Molchanov et al. [24] who propose an approach using a Recurrent 3D Convolutional Neural Network (R3DCNN). The proposed classifier has been trained and tested on the dataset, which has been collected by multiple sensors (i.e., SoftKinetic DS325 and DUO 3D) in the car simulator with both bright and artificial lighting. The SoftKinetic DS325 sensor enabled the acquisition of front-view color and depth videos. Additionally, the dense optical flow has been computed through the color videos, which allowed the acquisition of additional information. The DUO-3D sensor enabled the further acquisition of a pair of stereo IR-streams, which have been used to compute the IR-disparity map. The proposed approach has been tested on various data modalities, achieving 83.8% accuracy, when all data modalities have been used. Moreover, the proposed approach has been evaluated on two publicly available datasets, i.e., SKIG [81] and ChaLearn 2014 [82], achieving 98.6% and 98.2% accuracy, respectively. The proposed approach presents promising results; however, a 3D convolution is not currently supported by resource-constrained devices, e.g., Edge TPU and ARM microcontrollers.

Another interesting work related to the design of a highly performant classifier is [25]. This research does not directly deal with the problem of gesture recognition, but with the more general task of action recognition. This work proposes a novel classifier called Two-Stream Inflated 3D ConvNets (I3D). As the name implies, this topology builds upon state-of-the-art image classification architectures but inflates their filters and pooling kernels into a 3D structure. The proposed classifier has been tested against well-known action recognition datasets, thereby achieving 98.0% accuracy in the case of the UCF-101 dataset [83] and 80.9% accuracy in the case of the HMDB-51 dataset [84]. This work exhibits similar limitations to [24], namely a 3D convolution is not currently supported by devices with limited resources.

D’Eusanio et al. [26] propose a gesture recognition classifier based on Dense-161 architecture. The proposed system has been designed for the challenging automotive context, aiming at reducing the driver’s distraction during the driving activity. In this study [26], the proposed algorithm has been tested against two well-known datasets, i.e., the Briareo [80] and the Nvidia Dynamic Hand Gesture dataset [24], referred to as NVGestures. In the case of the Briareo dataset, the classifier has been tested on single data modalities and combinations of data modalities, i.e., RGB, infrared, and depth, thereby achieving in the best case 92% accuracy. With regards to the NVGestures dataset, the proposed classifier has been tested on single data modalities, i.e., RGB and depth, achieving in the best case 76.1% accuracy. The presented topology has 28 M parameters and requires 1 GB of GPU memory, in the unimodal setting. In the multimodal setting, the proposed model has about 56 M parameters and requires 2.7 GB of GPU memory. While the proposed approach presents an impressive performance, the hardware requirements do not allow the deployment on resource-constrained devices.

Another study proposing the FMCW radar-based gesture recognition system has been carried out by Wang et al. [85]. In this work, a method for continuous hand gesture recognition using an FMCW radar is proposed. First of all the 2-Dimensional fast Fourier transform (2D-FFT) is adopted to estimate the range and Doppler parameters. Then, the Multiple Signal Classification (MUSIC) algorithm is applied to estimate the angle of arrival of the hand towards the radar. A gesture detection method based upon the decision threshold is then used. Finally, the preprocessed gesture is used as input for the Fusion Dynamic Time Wrapping (FDTW) for classification. The proposed approach achieves 95.83% accuracy.

Another work dealing with radar-based gesture recognition has been proposed by Wang et al. [86]. This study concentrates on the exploration of this sensing modality, proposing a gesture processing scheme based on FFT and a deep learning classifier. The authors of this study propose a CNN–LSTM classifier trained and tested on the dataset consisting of 11 gestures. The proposed methodology achieved satisfying recognition accuracy of 87.17%.

Other studies dealing with radar-based gesture recognition have been proposed by Ritchie et al. [87,88]. In the first study [87], the authors introduce a database of radar micro-Doppler signatures called Dop-NET. This study checks the performance of several classifiers, i.e., fine tree, fine k-NN, linear discriminant, quadratic discriminant, SVM linear, and SVM quadratic achieving 69.7%, 71.4%, 54.6%, 59.7%, 61.9%, and 74.2% accuracy, respectively. In the next study [88], authors employ the k-NN classifier, thereby achieving 87.0% accuracy.

## 3. System Description and Implementation

In this section, we present the system components, evaluation methods, and implementation details (i.e., hardware details, operating parameters, experimental setup, proposed signal processing, and gesture vocabulary).

### 3.1. The General Overview of the Proposed Framework

**Figure 1:**
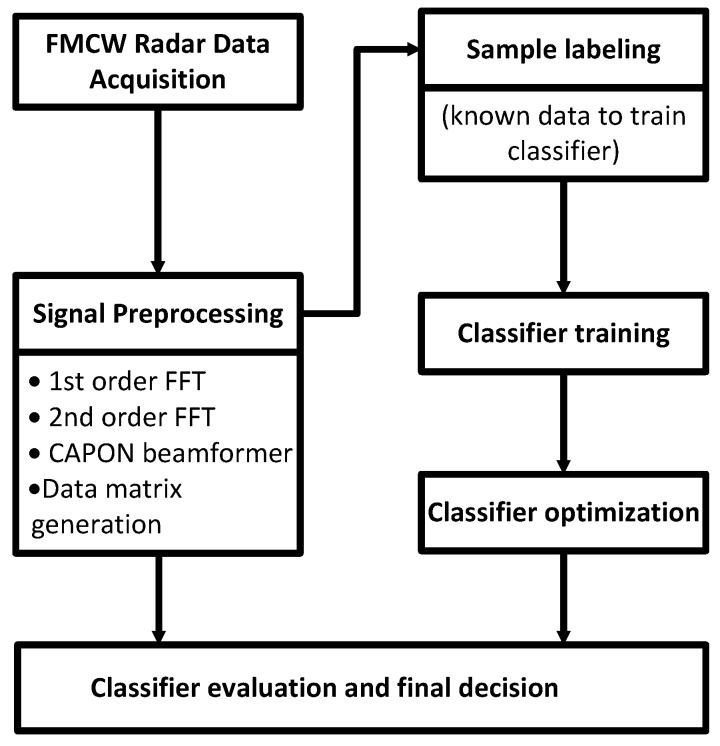
Data collection, preprocessing, training, and evaluation process of the proposed hand gesture recognition framework for FMCW radar.

Figure 1 presents the process of data collection, classifier training, and evaluation proposed in this study. Each sample has been first preprocessed and subsequently converted into the 3D tensor. After the training process, the model has been frozen, subsequently quantized in the post-training phase, compiled, and deployed on the Coral Edge TPU board.

### 3.2. Radar

**Figure 2:**
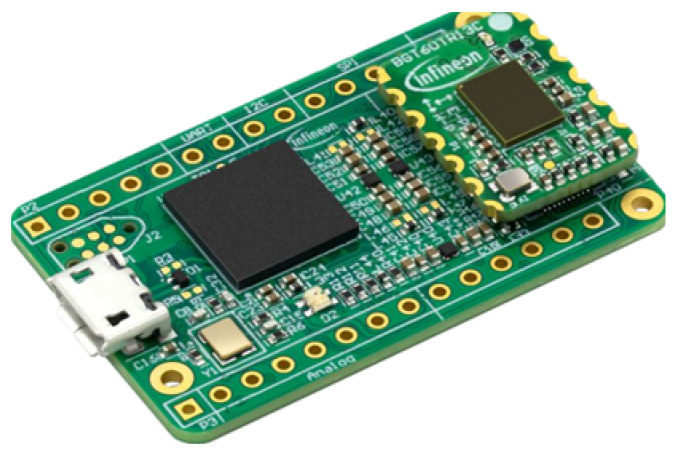
BGT60TR13C radar board [89].

The radar sensor used in this work is the BGT60TR13C FMCW radar sensor designed and manufactured by Infineon Technologies AG with the center frequency of 60.0 GHz. The BGT60TR13C is a low-power, low-cost, and high-resolution solution. The radar board has been depicted in Figure 2.

**Figure 3:**
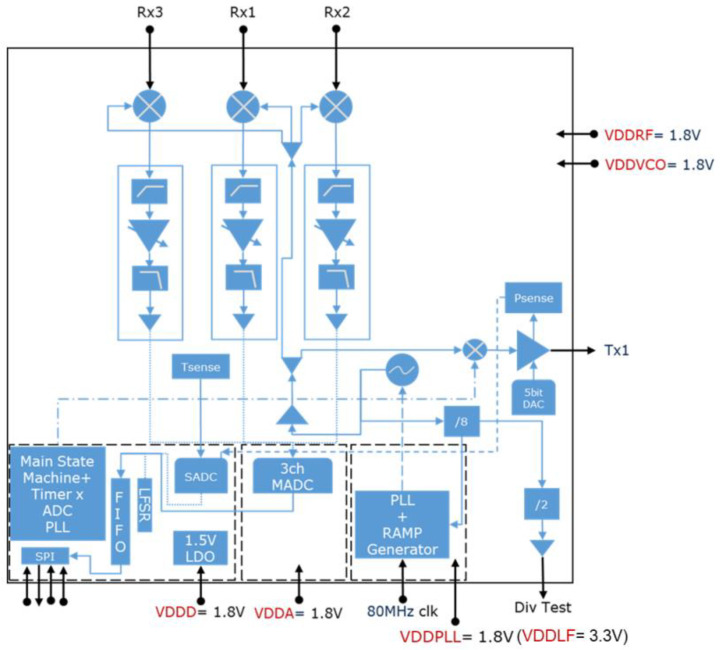
BGT60TR13C radar sensor block diagram [89]. The signal sensed by the three receiver channels (RX1, RX2, and RX3) is mixed with the transmitted signal from TX1, processed, and then converted digitally through the ADC.

The radar chip is equipped with three receiving antennas and one transmitting antenna. The operation principle of an FMCW radar sensor is as follows: the BGT60TR13C sends a periodic chirp signal through a transmitting antenna, and it receives a signal reflected from an object using one of the three receiving antennas with the round trip propagation delay $\tau_{k}$ and the Doppler shift $f_{D}$. Figure 3 represents the block diagram of the radar system.

**Figure 4:**
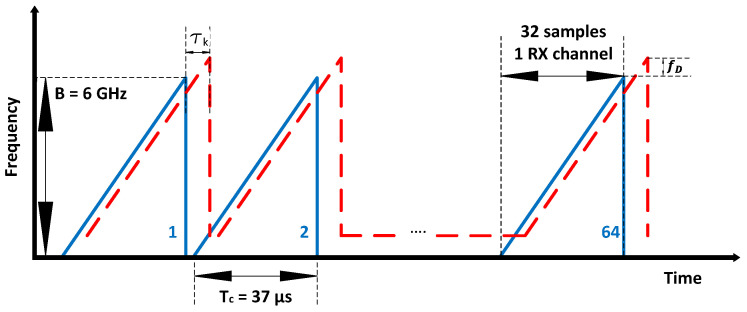
FMCW waveform in the frequency domain.

The transmitted and received signals are then mixed and passed to a baseband chain and to an analog-to-digital converter (ADC) with 12-bit resolution and up to 4 MSps sampling rate. Each baseband chain consists of a high pass filter, a voltage gain amplifier (VGA), and antialiasing filters. The digitized signal is stored in a FIFO buffer; then, the data is sent to an external host for further signal processing. This feature makes the device suitable for the hand gesture recognition application. The chipset transmits the signal up to 6 GHz (57 GHz–63 GHz) bandwidth; therefore, it provides the range resolution Δr of 2.5 cm and the velocity resolution Δv of 122 cm/s. Δr and Δv can be expressed with the following formulas:

(1) $$\Delta r=\frac{c}{2B}=2.5\;\mathrm{cm}$$

(2) $$\Delta v=\frac{c}{2f_{c}}\cdot \frac{1}{n_{c}T_{c}}=122\;\mathrm{cm}/s$$

where $f_{c}$ is the center frequency between 57 GHz and 63 GHz, which is set to 60 GHz, $T_{c}$ is the chirp duration, and $n_{c}$ is the number of repeatedly transmitted chirp signals, set to 37 μs and 64, respectively. The transmitted signal is modulated using the sawtooth wave function. Figure 4 presents the radar operating parameters.

### 3.3. Radar Signal Model

The frequency of the transmitted FMCW waveform with bandwidth *B* and chirp duration $T_{c}$ can be expressed as follows:

(3) $$f_{t}=f_{c}+\frac{B}{T_{c}}\cdot t$$

where $f_{c}$ is the carrier frequency. The reflected signal from the target is mixed with the replica of the transmitted signal resulting in beat signal. The phase of the beat signal after mixing due to *k*th point target is:

(4) $$\phi_{k}\left(t\right)=2\pi \left(f_{c}\tau_{k}+\frac{B}{T_{c}}t\tau_{k}-\frac{B}{2T_{c}}\tau_{k}^{2}\right)$$

The round trip propagation delay $\tau_{k}$ between the transmitted and received signal after reflection from the *k*th target with range $R_{k}$, radial velocity $v_{k}$, and speed of light *c*, approximately $3\times 10^{8}$ m/s, is expressed with the following formula:

(5) $$\tau_{k}=\frac{2\left(2R_{k}+v\right)}{c}$$

The intermediate frequency (IF) signal is the superposition of received signal from *K* point-scatters and expressed with the following formula:

(6) $$s_{IF}\left(t\right)=\sum_{k=1}^{K}\exp\left(2\pi \left(\frac{2f_{c}R_{k}}{c}+\left(\frac{2f_{c}v_{k}}{c}+\frac{2BR_{k}}{cT_{c}}\right)t\right)\right)$$

### 3.4. Radar Signal Processing

The collected radar raw signal is not easily interpretable; it is, in fact, hard to extract the relevant information from it, due to white noise and the influence of the environment surrounding the target. In the case of FMCW radar, waveforms expressed on the time-amplitude function are often not distinguishable.

#### 3.4.1. Range Doppler Image Generation

The radar signal processing consists of several steps. The frequency shifts due to range and velocity arising from multiple point targets at the IF signal are decoupled by generating a range-Doppler image (RDI) across three RX channels of the radar sensor. Denoting the time index *t* as $n_{i}$, where $n_{f}$ is the fast time index $0<n_{f}<T_{c}$, and $n_{s}$ as a slow time index. The received signal $s_{IF}\left(t;n_{k}\right)$ at frame $n_{k}$ forms the consecutive chirps arranged in the form of a 2D matrix, i.e., $s_{IF}\left(n_{s},n_{f};n_{k}\right)$. The RDI is generated for each channel by subtracting the mean value of each chirp from each sample, applying the Hann window function and zero padding. Then, the 1D fast Fourier transform (FFT) along the fast time direction resolves the signal in range, and the application of the Hann window function, zero padding, and 1D FFT along the slow time direction allows the extraction of the Doppler information. Subsequently, the absolute value of the two 1D FFT transforms is computed and the median and Wiener filters are applied to increase the signal-to-noise ratio. The ghost targets are removed by applying the OS-CFAR algorithm in both fast time and slow time directions. The two 1D FFTs transform the signal $s_{IF}\left(n_{s},n_{f};n_{k}\right)$, along fast time and slow time, into single RDI.

(7) $$S\left(p,q,n_{k}\right)=\sum_{n_{s}=1}^{Z_{N_{c}}}\left(\sum_{n_{f}=1}^{Z_{NTS}}w_{f}\left(n_{f}\right)s_{IF}\left(n_{s},n_{f};n_{k}\right)e^{\frac{-j2\pi pn_{f}}{Z_{NTS}}}\right)\cdot w_{s}\left(n_{s}\right)e^{\frac{-j2\pi qn_{s}}{Z_{N_{c}}}}$$

where NTS and $Z_{NTS}$ denote the number of transmitted samples and zero padding along fast time, respectively. $N_{c}$ and $Z_{N_{c}}$ stand for the number of chirps in a frame and zero padding along the slow time. $w_{f}\left(n_{f}\right)$ and $w_{s}\left(n_{s}\right)$ represent the window functions along fast time and slow time. *p* and *q* denote the index over the range and Doppler. RDI including the information about the range and radial velocity can be expressed as follows:

$$RDI=\left[\begin{array}{cccc} S(1,1) & S(1,2) & \cdots & S(Z_{NTS},1)\\ S(1,2) & S(2,2) & \cdots & S(Z_{NTS},2)\\ \vdots & \vdots & \ddots & \vdots \\ S(1,Z_{N_{c}}) & S(2,Z_{N_{c}}) & \cdots & S(Z_{NTS},Z_{N_{c}}) \end{array}\right]$$

#### 3.4.2. Angle Estimation

The next step of signal processing is the estimation of the direction of arrival (DOA). In our application, we implemented the minimum variance distortionless response (MVDR) or Capon beamformer [90]. The basic principle of digital beamforming is to scan the space by generating a maximum beam pattern corresponding to a selected direction and measuring the output power P(θ) of the digital signal $S(p,q,n_{k})$. The maximum power P(θ) corresponds to the DOA of the digital signal. The output power P(θ) is defined as follows:

(8) $$P\left(\theta \right)=w^{H}SS^{H}w=w^{H}R_{ss}w$$

where $R_{ss}$ is the covariance matrix, and *w* is the weight matrix.

The signal received from the antennas consists of the raw signal and noise. The raw signal for each channel is correlated since it comes from the same source. The noise is assumed to be uncorrelated Gaussian white noise; therefore, the covariance matrix of the noisy signal can be expressed as follows:

(9) $$R_{xx}=E\left\{s\left(t\right)s^{H}\left(t\right)\right\}$$

The goal of the Capon beamformer is minimizing the total variance under the constraint that the target response is unitary, hence Capon beamformer can be formulated as follows:

(10) $$min\left(P\left(\theta \right)\right)\;subject\;to\;w^{H}a\left(\theta \right)=1$$

where weight vector *w* can be written as follows:

(11) $$w=\frac{R_{ss}^{-1}a\left(\theta \right)}{a^{H}\left(\theta \right)R_{ss}^{-1}a\left(\theta \right)}$$

Substituting Equation (10) into (8), we obtain the equation for estimating the angle spectrum:

(12) $$P\left(\theta \right)=\frac{1}{a^{H}\left(\theta \right)R_{ss}^{-1}a\left(\theta \right)}$$

In our use case, the Capon beamformer is used for the azimuth angle estimation. The Capon beamformer for each frame generates a range-angle image (RAI).

#### 3.4.3. Dataset Generation

In this work, we apply a data transformation from a high-dimensional space into a low-dimensional space, to generate for every gesture range time, velocity time, and azimuth time maps. Generated RDIs and RAIs form the volume $S_{R}\in R^{t\times x\times y\times f}$ where t≥1. Each timestep stores an RDI and RAI denoted by $\Phi \in R^{x\times y\times f}$, where x×y correspond to the range and Doppler dimensions in the case of RDI, range and angle dimensions in the case of RAI, and *f* is the number of feature channels, which is in our case two, as the first channel stores an RDI, while the second RAI. Single RDI and RAI form a matrix with m×n dimensions, where x∈{1,..,m} and y∈{1,..,n}.

$$\Phi_{m,n}=\left[\begin{array}{cccc} a_{11} & a_{12} & \cdots & a_{1n}\\ a_{21} & a_{22} & \cdots & a_{2n}\\ \vdots & \vdots & \ddots & \vdots \\ a_{m1} & a_{m2} & \cdots & a_{mn} \end{array}\right]$$

**Figure 5:**
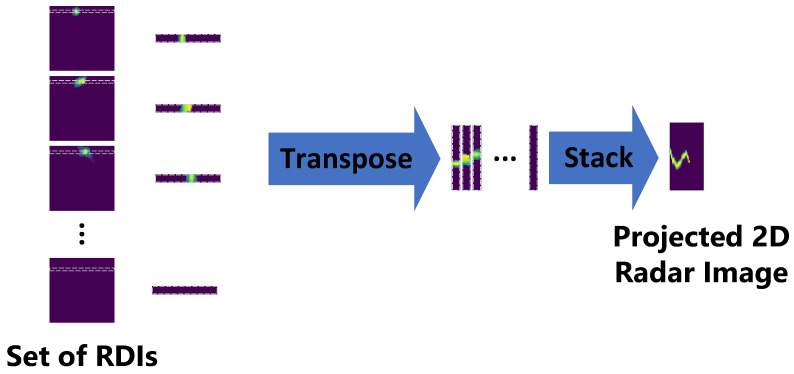
Projection of the extracted RDIs into 2D radar image.

The goal is to find an index (i,j) of the largest element $a_{i,j}^{max}$ in the matrix, denoting I={1,...,m} and J={1,...,n} as sets of row and column indices. There is an index i,j, ∃i∈I, and ∃j∈J such that $a_{ij}$ is the maximum element of the matrix. The next phase is slicing the vectors $R_{1\times n}^{t}$, $V_{n\times 1}^{t}$, and $A_{1\times n}^{t}$ with the vector representing the distance of the target from radar, radial velocity, and DOA in the given time step of the gesture. Subsequently, vectors $R_{1\times n}^{t}$ and $A_{1\times n}^{t}$ are transposed ${R_{1\times n}^{t}}^{T}$, ${A_{1\times n}^{t}}^{T}$. In the next step, vectors ${R_{1\times n}^{t}}^{T}$, $V_{n\times 1}^{t}$, and ${A_{1\times n}^{t}}^{T}$ are concatenated with the subsequent time slices forming range time, velocity time and azimuth time images. The proposed signal processing method enables the data dimensionality reduction, thereby leading to good classification results. Figure 5 depicts the generation of the range time image; however, the analogous procedure is applied generating velocity time and angle time images.

**Figure 6:**
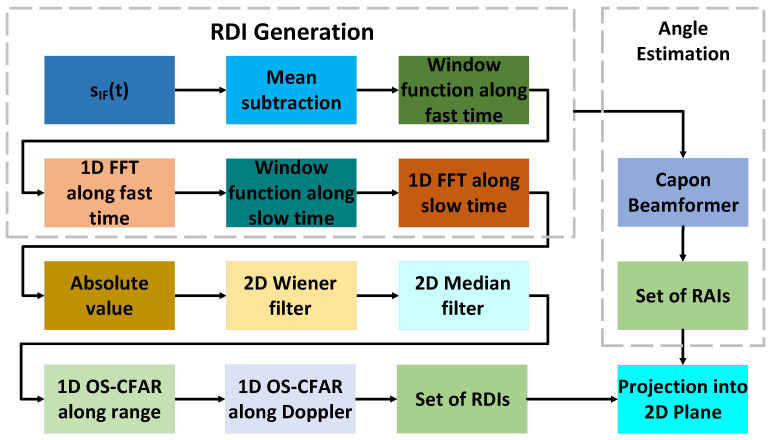
Generation scheme of range time, velocity time, and angle time images.

Figure 6 delineates the detailed generation scheme of range-time, velocity-time and angle-time images.

### 3.5. Gesture Vocabulary

**Figure 7:**
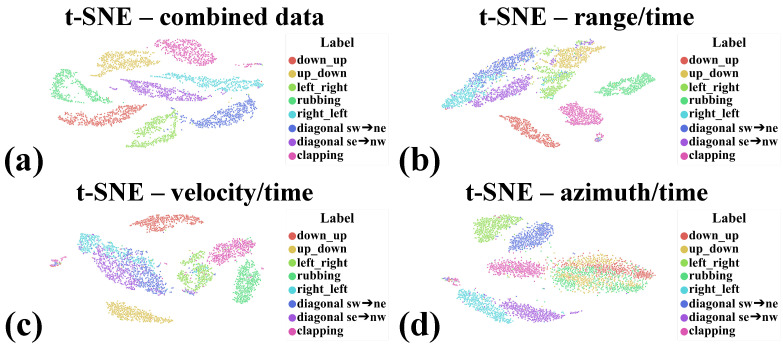
(**a**) t-SNE representation of all information, including range time maps, velocity time maps, and azimuth time maps. It is clearly visible that the composition of this information together allows for the separation of clusters; (**b**) t-SNE representation of range time maps; (**c**) t-SNE representation of velocity time maps; and (**d**) t-SNE representation of azimuth time maps.

The system defines eight gestures: ***(a) down -> up*** (swiping the hand from down to top), ***(b) up -> down*** (swiping the hand from top to bottom), ***(c) left -> right*** (swiping the hand from left to right), ***(d) rubbing (rubbing with fingers)***, ***(e) right -> left*** (swiping the hand from right to left), ***(f) diagonal southeast -> northwest*** (swiping the hand from left bottom corner to right top corner), ***(g) diagonal southwest -> northeast*** (swiping the hand from right bottom corner to left top corner), and ***(h) clapping*** (clapping hands). Figure 7 presents the t-SNE representation of the collected data. Figure 7 consists of subfigures (a), (b), (c), and (d) presenting the t-SNE representation of combined data, t-SNE representation of range time maps, t-SNE representation of velocity time maps, and t-SNE representation of azimuth time maps, respectively. It can be clearly noticed that concatenating the collected data, i.e., the composition of range time, velocity time, and azimuth time maps, allows for the best separation of clusters. Considering the remaining representations, we can notice that the quality of data separation is worse.

**Figure 8:**
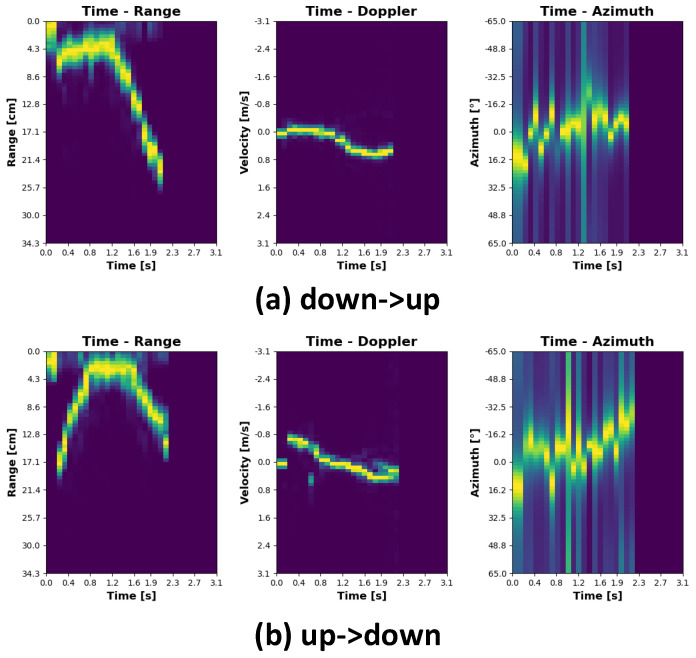
Gesture signatures.

**Figure 9:**
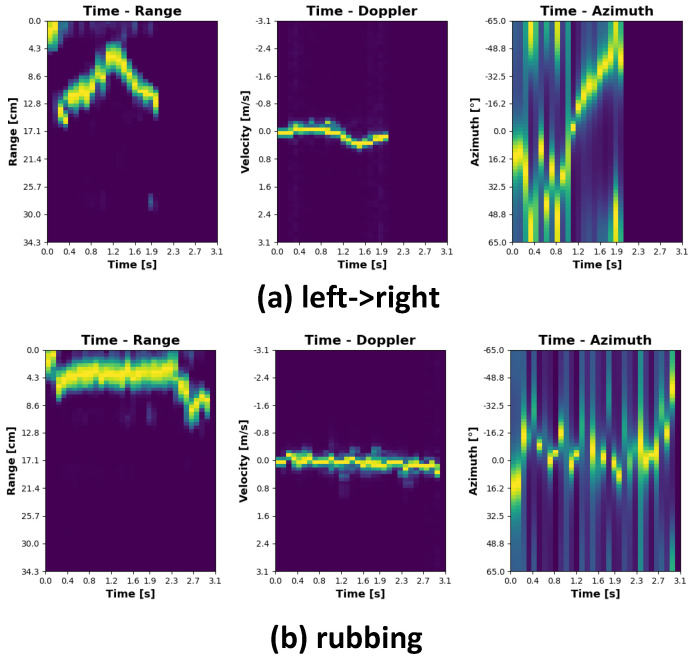
Gesture signatures cont.

**Figure 10:**
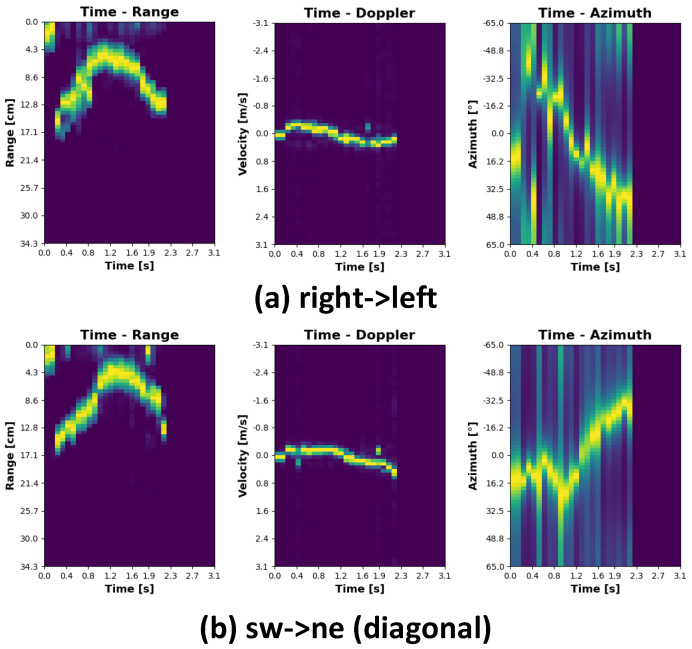
Gesture signatures cont.

**Figure 11:**
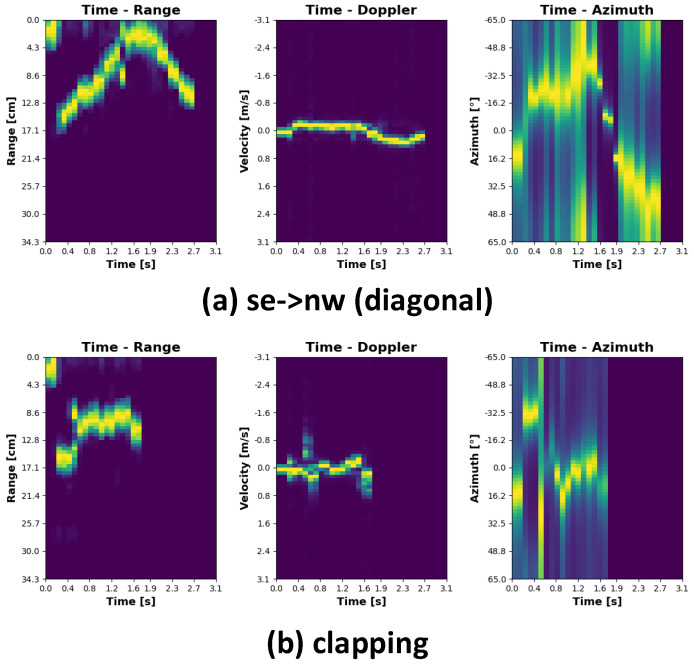
Gesture signatures cont.

The plots representing individual gestures have been depicted in Figure 8, Figure 9, Figure 10 and Figure 11. Every single gesture is represented by the range time, velocity time, and azimuth time maps. The gestures have been performed by five different individuals, within three days, in three different environments. None of the individuals have been previously trained on how to perform the gestures. The individual gestures in Figure 8, Figure 9, Figure 10 and Figure 11 are marked accordingly with the consecutive letters a–h. The temporal boundaries of gestures are based on a threshold mechanism. Every gesture is therefore sensed as long as the threshold is exceeded over time.

As can be noticed from the plots in Figure 8, Figure 9, Figure 10 and Figure 11, all the gestures differ from each other by some features in range, speed, or angle. By looking at the first two gestures, down-up and up-down, for example, the main differences lay in the range time plot. For the down-up gesture instance, the target, i.e., hand, is located in the early stage, approximately 3 cm above the radar. This trend is different for the up-down gesture, where in the early phase, the target is located around 20 cm above the sensor. In this case, range time and angle time maps exhibit similar behavior since the velocity in both cases is roughly the same, and on the horizontal plane, the angle practically does not change.

Regarding the gestures left -> right and right -> left, it can be noticed that the range time and velocity time maps exhibit similar tendencies; however, analyzing the angle time plots, it is clearly visible that the target approaches the sensor from the two opposite directions.

As for the rubbing gesture, the plots clearly show that the target’s distance from the sensor, the relative velocity, and the DOA roughly do not change.

By analyzing the southwest -> northeast (diagonal) and southeast -> northwest (diagonal) gestures, the range angle and velocity angle map results are very similar. However, the angle time plots show that the target approaches the radar from two different directions.

The plots representing the clapping gesture are slightly different from the others. While the distance from the sensor and the radial velocity does not change, the angle time map clearly shows a signal scattering pattern. It is explainable because range and velocity over time are relatively stable, while the target approaches the sensor from both sides, causing the signal scattering.

### 3.6. Experimental Setup

**Figure 12:**
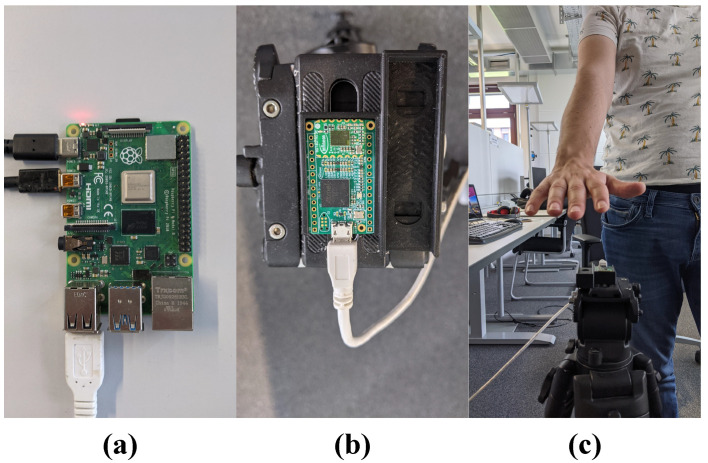
Data collection setup; (**a**) Raspberry Pi4; (**b**) 3D-printed case and radar board; and (**c**) tripod with 3D-printed case and radar board.

**Figure 13:**
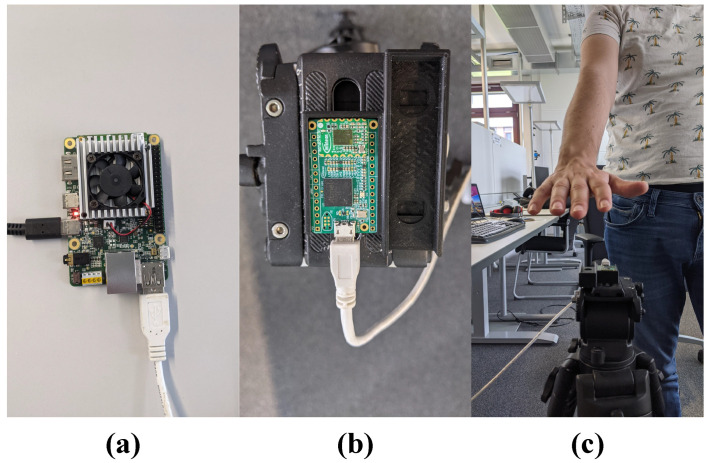
Inference setup; (**a**) Coral Edge TPU; (**b**) 3D-printed case and radar board; and (**c**) tripod with 3D-printed case and radar board.

The experimental setup consists of Raspberry Pi4, Coral Edge TPU accelerator, BGT60TR13C radar board, and a 3D-printed case, which is fixed to a camera tripod. The data collection software has been run on a Raspberry Pi4. However, the final, optimized model has been deployed on the Coral Edge TPU board. Figure 12 and Figure 13 present the data collection setup and inference setup.

## 4. Deep Learning Classifier

In this section, we present the details of the proposed deep neural classifier derived from MobileNetV1, which has been named Radar Edge Network. In the next subsections, we discuss the structural details of the proposed building blocks.

### 4.1. CNN Architecture

**Figure 14:**
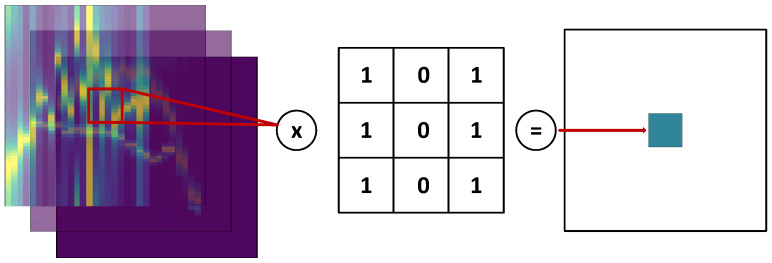
Convolution—principle of operation.

The typical CNN consists of the following building blocks:

- Input Layer: representing the input data in the form of a 3D tensor.
- Convolutional Layer: the main objective of a convolutional layer is the feature extraction achieved by convolving the input data with a kernel in the form of a 2D matrix. The filter kernels are moved through the input data generating the output (feature maps) of the convolutional layer. The principle of operation of the convolutional layer is depicted in Figure 14.
- Batch Normalization Layer: the layer used after convolution to speed up the training process.
- Activation Function: the activation function, e.g., ReLu, LeakyRelu, ReLu6, SiLu, SeLu, and GELU. It is used to introduce the nonlinearity, and to be able to learn more sophisticated data patterns.
- MaxPooling2D: the layer utilized for the dimensionality reduction and feature extraction of the most relevant data.
- Regularization Layers: e.g., Dropout, AlphaDropout, and GaussianDropout; employed to make the classifier noise resistant.

### 4.2. Radar Edge Network

The operations discussed above represent the typical structure of CNN architecture. Typically the layers are stacked on each other forming the hidden layer of a CNN. The gradual increment of the number of layers and number of the convolutional filters is the common way of increasing the complexity of feature extraction in the network, thereby contributing to higher classification accuracy. The increase of the number of layers generates some limitations, namely the networks can become vulnerable to overfitting problems, and the increased number of parameters prevents the model from deployment on edge computing devices. This work presents the novel building block—Depthwise Expansion Module derived from MobileNetV1 topology, which is commonly used in applications related to edge computing. The proposed building block is based on the main building block of MobileNetV1—depthwise separable convolutions. The proposed structural-level amendments enable the extraction of the most relevant features while saving a significant number of parameters, thereby making the network less prone to overfitting problems. A detailed description of the proposed block and the proposed model is presented in the next sections.

#### 4.2.1. Depthwise Separable Convolutions

**Figure 15:**
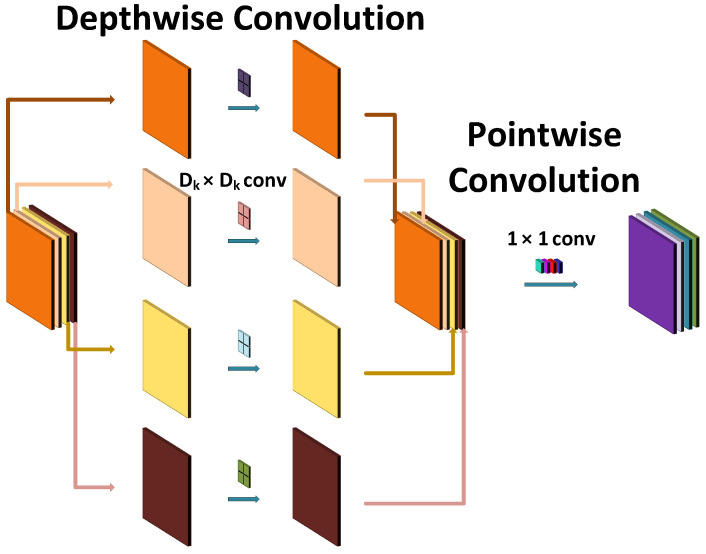
Depthwise separable convolution—principle of operation.

The building block of MobileNetV1 is a depthwise separable convolution. The main advantage of depthwise separable convolution is the drastic reduction of the number of parameters achieved by applying a depthwise convolution and a 1×1 convolution called a pointwise convolution. As depicted in Figure 15, the depthwise convolution applies a single kernel to each input channel (channelwise), while the standard convolution applies the single filter to each input channel. The computational cost of standard convolution can be expressed as follows:

(13) $$D_{K}\cdot D_{K}\cdot M\cdot N\cdot D_{F}\cdot D_{F}$$

where *M* is the number of input channels, $D_{F}$ is the spatial dimension height and width of the input feature map, *N* is the number of output channels, and $D_{K}$ is the spatial dimension height and width of the kernel. While a pointwise convolution has the following computational cost:

(14) $$D_{K}\cdot D_{K}\cdot M\cdot D_{F}\cdot D_{F}$$

The combination of depthwise convolution and pointwise convolution is called a depthwise separable convolution. The computation cost of depthwise separable convolution is expressed as follows:

(15) $$D_{K}\cdot D_{K}\cdot M\cdot D_{F}\cdot D_{F}+M\cdot N\cdot D_{F}\cdot D_{F}$$

The reduction in computation is as follows:

(16) $$\frac{D_{K}\cdot D_{K}\cdot M\cdot D_{F}\cdot D_{F}+M\cdot N\cdot D_{F}\cdot D_{F}}{D_{K}\cdot D_{K}\cdot M\cdot N\cdot D_{F}\cdot D_{F}}=\frac{1}{N}+\frac{1}{D_{K}^{2}}$$

#### 4.2.2. Depthwise Expansion Module

The proposed building block is inspired by the MobileNetV1. In the original MobileNetV1 implementation, the standard CNN2D and Depthwise2D convolutions are interleaved with each other, increasing linearly the number of convolutional filters, thereby causing a drastic increment in the number of parameters.

**Figure 16:**
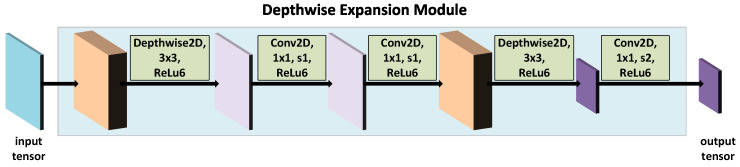
Depthwise expansion module.

In this work, we propose a module—Depthwise Expansion, employing the bottleneck approach, i.e., it makes use of the Depthwise2D convolution to increase the number of feature maps, followed by standard CNN2D, which performs the final feature embedding. First, the Depthwise2D convolution with a double number of feature maps is applied. This is achieved by setting the depth multiplier parameter to 2. The Depthwise2D convolution is followed by a standard CNN2D, decreasing by half the number of feature maps and performing the most relevant feature embedding. Subsequently, another CNN2D is applied to perform further feature extraction. The number of CNN2D filters is changed according to the following rule: 2·⌊α·filters⌋, where filters for the first Depthwise Expansion module is 64, while for the second Depthwise Expansion module it is 32. α is the parameter determining the depth of the network and its values are as follows: 1, 0.75, 0.50, and 0.25. The extracted features are fed to the second Depthwise2D convolution which doubles the number of generated feature maps. Finally, the standard CNN2D with stride 2 and kernel size 1×1 is applied for feature embedding and spatial dimensionality reduction. Figure 16 presents the proposed module.

#### 4.2.3. Proposed Classifier

As stated earlier, the Radar Edge Network is based on an architecture presented by Google, named MobileNetV1. In the original implementation, Google linearly increases the complexity of the network by incrementing the number of convolutional filters. MobileNetV1 applies 13 depthwise separable convolutional modules, followed by global average pooling for a drastic dimensionality reduction, and a fully connected layer performing the final classification. Although the base MobileNetV1 architecture is small and offers low latency capabilities, Google introduced a very simple parameter α called width multiplier. This parameter is used to construct a smaller and less computationally expensive model, manipulating the number of generated feature maps at each layer. The parameter α∈(0,1], and its values are as follows: 1, 0.75, 0.50, and 0.25. The α=1 is the baseline MobileNetV1 and α<1 are reduced MobileNets.

As opposed to the original MobileNetV1 implementation, the proposed classifier does not apply an incremental approach but increases the number of feature maps applying the Depthwise2D convolution. Then the number of feature maps is decreased by performing the feature embedding. Instead of global average pooling, a standard flattening layer is applied.

**Figure 17:**
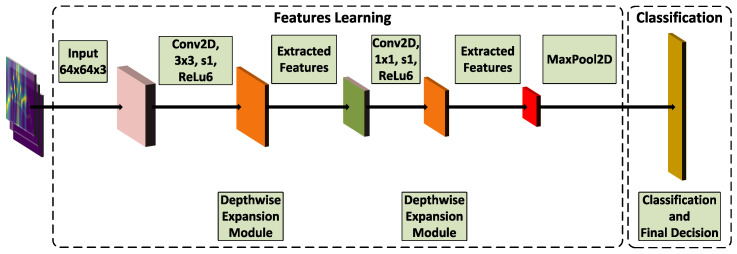
Proposed classifier.

Finding the best set of parameters is usually a very complex problem, and it is typically strictly task dependent. In this work, we tested several possible variants of Radar Edge Network with different values of α parameters, i.e., 0.25, 0.50, 0.75, and 1.00. We conducted a detailed analysis of the relationship between accuracy and the number of depthwise expansion modules, model size and the number of depthwise expansion modules, number of depthwise expansion modules and inference time, and model size and inference time. The accuracy as a function of the number of the depthwise expansion modules was considered, and the network with the highest accuracy is presented in Figure 17.

First, the raw radar signal is preprocessed, then the 3D input tensor is constructed, i.e., the range time, Doppler time, and azimuth time images are fed to the deep neural classifier. The Radar Edge Network consists of two convolutional layers and two depthwise expansion modules, followed by a MaxPooling2D layer, flattening layer, and fully connected layer performing the final classification. The name depthwise expansion refers to the application of the depthwise convolution to increase the number of extracted features. The standard convolution is applied to drastically reduce the number of feature maps. To the best of our knowledge, this type of module has never been implemented in the field of gesture recognition with radar. As stated, we tested several variants of the proposed network with parameter α varying from 0.25 to 1.0. α∈(0,1].

### 4.3. Edge TPU Deployment

**Figure 18:**
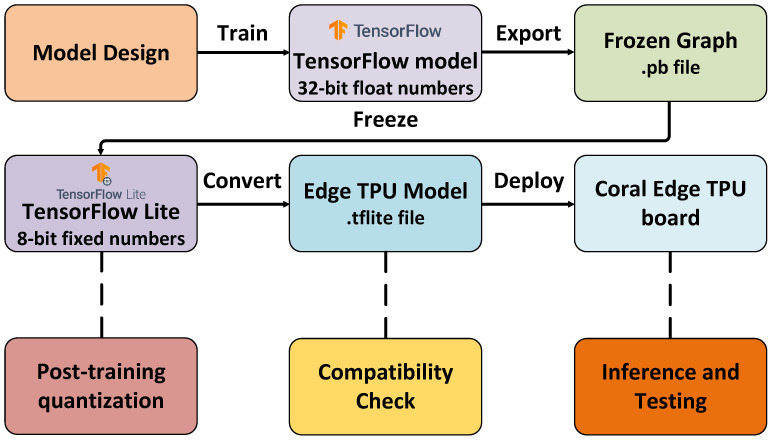
Edge TPU deployment workflow diagram.

In this section, we describe the steps taken to deploy the model on the Coral Edge TPU board. In the first stage of the deployment process, the model is implemented and trained. Then, the weights are converted to constants and the model is optimized, i.e., quantized to 8-bit integer accuracy. In this work, we perform the post-training quantization using the representative dataset. The model is compiled in a binary format supported by the Edge TPU and a compatibility check is performed, i.e., execution compatibility on the TPU chip. Finally, the compiled model is deployed on the Edge TPU board and the inference and performance tests are performed. Figure 18 presents the Edge TPU deployment workflow.

## 5. Performance Evaluation

In this section, we present and discuss the experimental results. First, we analyze the test accuracy of the proposed classifiers. Then, we compare the performance of the proposed classifiers with the existing techniques, i.e., we analyze the test accuracy achieved by the classifiers deployed on the x86 and Coral Edge TPU platforms. Next, we investigate the model sizes for both implementations, i.e., x86 and Coral Edge TPU. Then, we consider and compare the inference times attained for both implementations, i.e., x86 and Coral Edge TPU. Finally, we discuss the results, and we compare the performance of the proposed classifiers with classifiers widely used in the edge computing field.

### 5.1. Classification Accuracy

**Figure 19:**
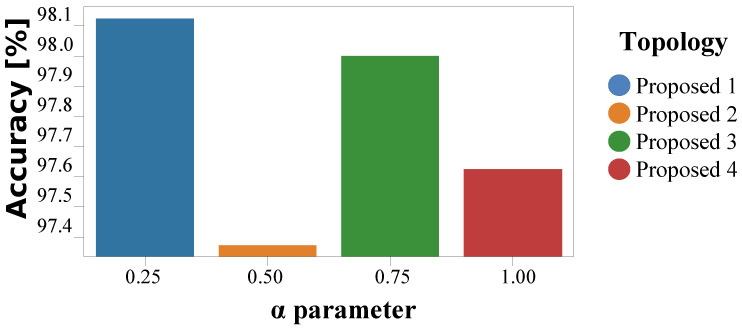
Test accuracy for different values of alpha parameters for the classifier.

We performed several structural adaptations while designing the deep learning topology for hand gesture recognition. To determine the most optimized model, we trained several models dependent on an α parameter which defines the number of feature maps per CNN2D layer. The proposed topologies with the increasing value of α∈{0.25,0.50,0.75,1.00} have been called Proposed 1, Proposed 2, Proposed 3, and Proposed 4. The accuracy as a function of different values of α parameter is depicted in Figure 19. The vertical axis represents the accuracy, while the horizontal axis represents the classifiers with different values of α parameter.

It can be observed through the bar plots in Figure 19 that the networks with a value of α parameter equal to 0.25 achieved the best accuracy (98.13%). As illustrated in Figure 19, the topology with the lowest value of α parameter achieves the best convergence to the dataset. In addition, the topologies with increasing α parameter slightly deteriorate the classification accuracy.

**Figure 20:**
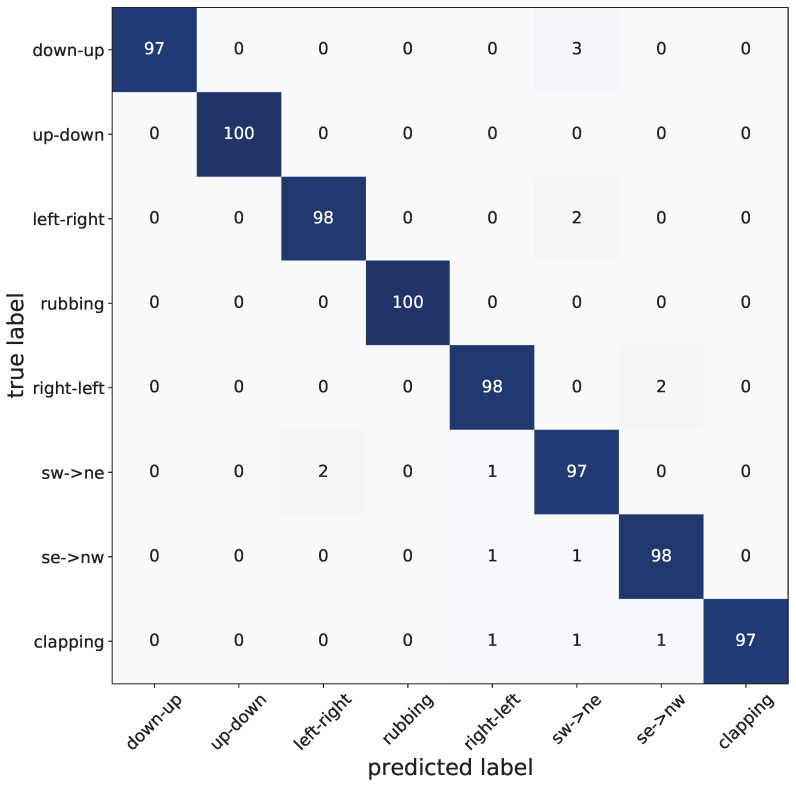
Confusion matrix.

Figure 20 displays the confusion matrix of the proposed gesture recognition framework. The rows represent the original gesture class, whereas the columns present the predicted gesture class. The classification accuracy of each gesture is presented in yellow in the main diagonal, whereas the erroneously classified gestures are shown in dark violet. As can be seen, the up-down and rubbing gestures show a higher accuracy, as they generate highly distinguishable patterns in comparison to the other gestures. The remaining hand gestures exhibit a slightly lower accuracy rate compared to up-down and rubbing. Their misclassification rate oscillates in fact, between 2% and 3% more, mainly due to their more complicated patterns.

### 5.2. Comparison with Existing Techniques

In this section, we carry out a detailed analysis of the performance, including accuracies, model sizes, and inference times. First, we compare the proposed topology with a classic CNN3D architecture, consisting of four CNN3D layers, which is trained from scratch. The further comparisons include the CNN2D and the MobileNetV2 with a variable number of bottleneck modules. The traditional CNN2D classifier consists of seven layers and it has also been trained from scratch. Table 1 presents the test accuracies of non-optimized and optimized classifiers. It can be seen, in the case of implementation on x86 processor as well as on the Edge TPU, that the classification accuracies dwell on similar levels. The best accuracy is achieved by the CNN3D classifier. In the case of the deployment on an x86 processor, the CNN3D achieves 99.63% accuracy, while the classification for Edge TPU is not feasible due to the lack of 3D CNN support. The lowest accuracy is achieved with the standard CNN2D methods. In both cases, the test accuracy remains on a comparable level, i.e., the models achieve 86.25% and 85.88% for x86 and Edge TPU implementation, respectively. The other classifiers in terms of classification accuracy remain on relatively the same level.

Table 2 presents the comparison of our proposed methods with other gesture recognition approaches. The table provides information about the model, the number of recognized gestures, the test accuracy, and the type of algorithm, i.e., deep learning, FDTW, k-NN, linear discriminant analysis (LDA), quadratic discriminant analysis (QDA), or support vector machine (SVM). We can see that the deep learning methods dominate among the gesture recognition algorithms. In most of the cases, the deep learning methods are superior to the standard methods. It is particularly noticeable in the experiments carried out by Ritchie et al. [87]. In this work, a radar micro-Doppler database representing four gestures is introduced. The proposed database has been used for the training of several classifiers, i.e., k-NN, LDA, QDA, and SVM, achieving relatively average accuracy results. Further, the presented results do not allow a real-time system operation. In the next work, Ritchie et al. [88] carried out a feature extraction, obtaining the following features: spectrogram summed intensity, spectrogram variance, spectrogram mean power, singular value decomposition (SVD) of spectrogram, and entropy of spectrogram intensity. The obtained features allowed achieving an accuracy of 87% with the k-NN classifier. It should be noticed that the classifiers used by Ritchie et al. [87,88] are not directly supported by edge devices. The other work addressing the gesture recognition problem was carried out by Lien et al. [77]. This work introduces the radar as a novel sensing modality, which can be used for gesture recognition. In this study, the gesture recognition procedure is realised employing the random forest classifier, which achieves 92.10% accuracy. In this case, the employed classifier is not also directly supported by the random forest classifier. Wang et al. [85] proposes the radar-based gesture recognition system. In this case, the system supports the recognition of six gestures. This work introduces the non-deep learning approach based on an FDTW algorithm, achieving 95.83% accuracy. The remaining approaches are based on the deep-learning techniques; however, only two of them provide the support for edge computing devices, i.e., the proposed method and [79].

One very important parameter in the case of deployment on resource-constrained devices is the model size. Table 3 presents the model sizes for the deployment on both the x86 processor and the Edge TPU. It can be seen that the CNN3D generates a large number of parameters, which leads to a large model size of around 12 MB, and thereafter it does not enable the deployment on resource-constrained hardware. In the case of the other classifiers, a significant difference in model size between the non-optimized and the optimized versions can be noticed. The smallest model size has been achieved by the CNN2D classifier, where the sizes for the non-optimized and optimized versions are 375.89 KB and 80.67 KB, respectively. Regarding the MobileNetV2 classifier, the model sizes for the x86 processor are 1770.96 KB, 2028.85 KB, 2287.06 KB, 2545.35 KB, 2804.27 KB, and 3063.25 KB, whereas the model sizes for Edge TPU implementation are 200.67 KB, 232.67 KB, 264.67 KB, 296.67 KB, 328.67 KB, and 360.67 KB. The best compression results have been achieved in the case of the proposed model. The model sizes for x86 implementation are 624.92 KB, 999.00 KB, 1543.89 KB, and 2233.44 KB, while the model sizes for Edge TPU implementation are 92.67 KB, 140.67 KB, 220.67 KB, and 280.67 KB.

The last analyzed parameter is the mean inference time. This parameter strongly influences the interaction experience, which plays a particular role during real-time system operations. Table 4 presents the achieved inference times for the deployment on the x86 and Edge TPU. Analyzing the data in Table 4, one may notice the benefits coming from the Edge TPU implementation. It can be seen that in most cases the inference times for x86 implementation are significantly longer, i.e., 3.57 ms, 1.16 ms, 2.19 ms, 4.17 ms, 5.66 ms, 8.52 ms, 8.74 ms, 10.42 ms, 5.74 ms, 10.18 ms, 14.22 ms, and 20.73 ms. Edge TPU implementations show significantly shorter inference times, i.e., 3.61 ms, 1.19 ms, 1.52 ms, 1.65 ms, 1.79 ms, 1.92 ms, 2.04 ms, 1.28 ms, 1.63 ms, 1.76 ms, and 1.90 ms.

## 6. Conclusions

In this work we have presented a novel deep learning classifier—Radar Edge Network. We have illustrated the detailed implementation of a hand gesture recognition system using an FMCW radar. The Radar Edge Network introduces the deep learning module—Depthwise Expansion Module inspired by MobileNetV1 architecture. Essentially, the proposed module employs the Depthwise2D convolution followed by the traditional CNN2D to perform the feature extraction. The application of Depthwise2D convolution has several benefits. Namely, it allows for saving a significant number of parameters, which then has an advantageous effect on the model size and the deployment on the edge. The proposed module increases the number of extracted feature maps using the Depthwise2D convolution and then employs the standard CNN2D with a 1 × 1 filter size for feature embedding. Then, the Depthwise2D convolution doubles the number of feature maps, and CNN2D with 1 × 1 filter size performs the final feature embedding.

Additionally, the proposed signal processing approach leads to the decreasing of data dimensionality. This is of particular importance in the case of the deployment on resource-constrained devices. Furthermore, thanks to the simplified data shape, it is possible to design a model that achieves very good classification performance while being also supported by edge computing systems.

Moreover, this work analyzes the effect of weight quantization and, to the best of our knowledge, proposes the first 8-bit integer implementation of the radar-based gesture recognition system deployed on the edge device such as Edge TPU. The results presented above validate our solution, particularly in terms of test accuracy, model size, and inference time. Additionally, we carried out a rigorous comparison with the state-of-the art gesture recognition approaches. Table 1 presents the classification results. It can be seen that the best classification result has been achieved by the CNN3D classifier. However, the CNN3D operation is not supported on resource-constrained devices, e.g., Edge TPU. In addition, taking a closer look at Table 3, it can be noticed that the model size of CNN3D is around 12 MB. This feature is another important factor that does not permit a constrained edge implementation. In the case of the remaining classifiers, the classification results are slightly worse; however, the difference is not very significant, i.e., in most cases, the classification results remain on a similar level. Analyzing Table 3, we can observe that in the case of x86 implementation, the model sizes are significantly larger and that the 8-bit integer implementation enables a significant amount of memory saving. A similar tendency can be observed with inference times. The optimized versions of classifiers offer significantly shorter inference times than in the case of x86 versions. It allows us to confirm the validity of our optimizations.

Table 2 presents the performance of various gesture approaches, not limited to deep learning and radar-based approaches. It consists of four columns representing the reference to the model, number of recognized gestures, the achieved accuracy, and type of algorithm. It can be seen that deep learning techniques are the most significant part of gesture recognition solutions. Table 2 reports also the non-deep learning approaches. In most cases, the non-deep learning approaches do not offer sufficient performance for real-time system operation. Regarding the non-deep learning approach, Wang et al. [85] propose in their work the system supporting six gestures and achieving 95.83% accuracy, based on an FDTW algorithm. The deep learning approaches are very often leading to superior results in comparison to the standard approaches. The performance of the classifier is also strictly dependent on the dataset complexity. It is particularly visible in the cases of the following studies [23,24,26]. Moreover, the dataset structure imposes the high complexity of the classification algorithm. In our case, we ease the dataset structure to save hardware resources and to be able to design a less complex classifier.

As future work, we will develop the software allowing for the data transfer between the radar board and the Coral Edge TPU board, then we will design a real-time version of our system to construct a standalone hardware and software solution. Moreover, in order to test the robustness of the proposed classifier, we will record the test dataset in several different environments. 

## Figures and Tables

**Table 1:** Comparative characteristics of accuracies for the non-optimized and the optimized versions.

		Accuracy [%]	
		**x86**	**Edge TPU**
**Topologies**	CNN3D	99.63%	N/A
	CNN2D	86.25%	85.88%
	MobileNetV2—1 bottleneck	98.88%	98.88%
	MobileNetV2—2 bottleneck	99.00%	98.75%
	MobileNetV2—3 bottleneck	97.13%	97.25%
	MobileNetV2—4 bottleneck	98.50%	98.50%
	MobileNetV2—5 bottleneck	97.75%	97.75%
	MobileNetV2—6 bottleneck	98.00%	97.88%
	**Proposed 1**	98.00%	98.13%
	**Proposed 2**	97.50%	97.38%
	**Proposed 3**	98.13%	98.00%
	**Proposed 4**	97.63%	97.63%

**Table 2:** Comparison with other approaches. DL: deep learning, k-NN: k-Nearest Neighbour, LDA: linear discriminant analysis, QDA: quadratic discriminant analysis, SVM-l: support vector machine with linear kernel, SVM-q: support vector machine with quadratic kernel.

Model	No. Gestures	Accuracy	Type of Algorithm
Hazra et al. [27]	5	94.34%	DL
Zhang et al. [74]	8	96.00%	DL
Ahmed et al. [75]	8	95.00%	DL
Hazra et al. [28]	6	94.50%	DL
Molchanov et al. [76]	11	94.10%	DL
Lien et al. [77]	4	92.10%	RF
Chmurski et al. [78]	4	95.05%	DL
Chmurski et al. [79]	4	98.10%	DL
D’Eusanio et al. [23]	25	87.60%	DL
D’Eusanio et al. [23]	12	97.20%	DL
Molchanov et al. [24]	25	83.80%	DL
D’Eusanio et al. [26]	25	76.10%	DL
D’Eusanio et al. [26]	12	92.00%	DL
Wang et al. [85]	6	95.83%	FDTW
Wang et al. [86]	4	87.17%	DL
Ritchie et al. [87]	4	69.7%	DT
Ritchie et al. [87]	4	71.4%	k-NN
Ritchie et al. [87]	4	54.6%	LDA
Ritchie et al. [87]	4	59.7%	QDA
Ritchie et al. [87]	4	61.9%	SVM-l
Ritchie et al. [87]	4	74.2%	SVM-q
Ritchie et al. [88]	4	87.0%	k-NN
**Proposed 1 (Edge TPU)**	8	98.13%	DL

**Table 3:** Comparative characteristics of model sizes for the non-optimized and the optimized versions.

		Size [KB]	
		**x86**	**Edge TPU**
**Topologies**	CNN3D	12,586.58	N/A
	CNN2D	375.89	80.67
	MobileNetV2—1 bottleneck	1770.96	200.67
	MobileNetV2—2 bottleneck	2028.85	232.67
	MobileNetV2—3 bottleneck	2287.06	264.67
	MobileNetV2—4 bottleneck	2545.35	296.67
	MobileNetV2—5 bottleneck	2804.27	328.67
	MobileNetV2—6 bottleneck	3063.25	360.67
	**Proposed 1**	624.92	92.67
	**Proposed 2**	999.00	140.67
	**Proposed 3**	1543.89	220.67
	**Proposed 4**	2233.44	280.67

**Table 4:** Inference time.

		Inference [ms]	
		**x86**	**Edge TPU**
**Topologies**	CNN3D	3.57	N/A
	CNN2D	1.16	3.61
	MobileNetV2—1 bottleneck	2.19	1.19
	MobileNetV2—2 bottleneck	4.17	1.52
	MobileNetV2—3 bottleneck	5.66	1.65
	MobileNetV2—4 bottleneck	8.52	1.79
	MobileNetV2—5 bottleneck	8.74	1.92
	MobileNetV2—6 bottleneck	10.42	2.04
	**Proposed 1**	5.74	1.28
	**Proposed 2**	10.18	1.63
	**Proposed 3**	14.22	1.76
	**Proposed 4**	20.73	1.90

## Data Availability

The data presented in this study are available on request from the corresponding author. The data are not publicly available due to internal company board policy.

## Abbreviations

The following abbreviations are used in this manuscript:

HCI	Human–Computer Interaction
FMCW	Frequency-Modulated Continuous Wave
RGB	Red Green Blue
ToF	Time of Flight
3DCNN	3D Convolutional Neural Networks
LSTM	Long Short-term Memory
RNN	Recurrent Neural Networks
IoT	Internet of Things
LRACNN	Long Recurrent All Convolutional Neural Network
NCS 2	Neural Compute Stick 2
RDM	Range-Doppler Map
k-NN	k-Nearest Neighbour
RFC	Random Forest Classifier
CWT	Continuous Wavelet Transform
R3DCNN	Recurrent 3D Convolutional Neural Network
I3D	Inflated 3D ConvNets
2D-FFT	2-Dimensional Fast Fourier Transform
MUSIC	Multiple Signal Classification
FDTW	Fusion Dynamic Time Wrapping
LDA	Linear Discriminant Analysis
QDA	Quadratic Discriminant Analysis
SVM	Support Vector Machine
ADC	Analog to Digital Converter
VGA	Voltage Gain Amplifier
RDI	Range-Doppler Image
FFT	Fast Fourier Transform
RAI	Range-Angle Image
SVD	Singular Value Decomposition
MVDR	Minimum Variance Distortionless Response
DOA	Direction of Arrival
